# The effect of chemotherapy regimens in male germ cell tumors on the development of primary hypogonadism

**DOI:** 10.1038/s41598-024-78765-w

**Published:** 2024-11-15

**Authors:** Analena Handke, Marla Geller-von Bargen, Aykhan Isgandarov, Mulham al Nader, Ulrich Krafft, Christopher Darr, Boris Hadaschik, Viktor Grünwald, Lukas Püllen

**Affiliations:** 1 Department of Urology, University of Duisburg-Essen and German Cancer Consortium (DKTK)-University Hospital Essen, Essen, Germany; 2 Department of Urology, Marienhospital Herne, Ruhr-University Bochum, Herne, Germany; 3 Clinic for Medical Oncology and Department of Urology, West-German Cancer Center, University Hospital Essen, Essen, Germany

**Keywords:** Germ cell tumor, Secondary hypogonadism, Medical research, Urology, Diseases, Urogenital diseases

## Abstract

Male germ cell tumors (GCT) have excellent survival. Long-term sequelae in cancer survivors are an evolving field. We evaluated the risk of patients with GCT to develop primary hypogonadism and adherence to guideline-recommended therapy in a real-world cohort. Monocentric study at a tertiary cancer centre to evaluate treated GCT-patients (2001–2019). Post therapeutic male endocrine function, International Index of Erectile Function (IIEF)-5 and The aging males’ symptoms rating scale (AMS) questionnaires were assessed. The overall response rates were low, with 44 of 402 contacted patients participating in the study. From these, 32(73%) underwent blood analysis, 42(95%) answered the IIEF-5 and 43(98%) the AMS. Latent hypogonadism (serum testosterone 8–12 nmol/l) was found in *n* = 9 (28%) and manifest hypogonadism (testosterone < 8 nmol/l) in *n* = 8 (25%). 50% (*n* = 21) indicated erectile dysfunction on IIEF-5 (cut off ≤ 21 pts.) and 62.8% (*n* = 27) reported symptomatic affection on AMS (cut off ≥ 27 pts.). Majority of tested patients revealed different degrees of hypogonadism. Standard instruments were able to detect gonadal damage in > 50%, which underscored the clinical need to evaluate endocrine function in cancer survivors. We further indicated the difficulties of today’s research and provided starting points to assess barriers for study participations.

## Introduction

The favourable cure rates render treatment of childhood and young adult cancer patients of specific interest in regard to the occurrence of chronic and long-term adverse effects in cancer survivors^1^. Germ cell tumors (GCT) in men occur most frequently between 20 and 40 years, with a relative 5-year overall survival of 96% for localized disease, and for metastasised disease 67–86%, depending on prognosis group^2–4^. In patients with metastatic spread, risk-adapted chemotherapy with cisplatin and etoposide with or without bleomycin (BEP) or ifosfamide (VIP) are considered standards of care^5,6^.

Hypogonadism is a long-term adverse effect of different chemotherapy regimens and therapy intensities in GCT. 11–34% of patients develop hypogonadism with an association of higher rates for fatigue, affection on the ability to father children, the development of cardiovascular disease, osteoporosis, metabolic syndrome, type 2 diabetes and in general worse health and reduced quality of life^7–11^. There is a medical need to better understand the adverse effects of chemotherapy on gonadal function in GCT patients^6,12^. To our knowledge, no appropriate preventive therapy exists currently to attenuate long-term hypogonadism. Furthermore, diagnostic instruments for objective symptom assessment are lacking.

The aim of this study is to investigate to what extent the use of GCT therapies have an additional long-term effect due to their gonadal damage potential with regard to the development of hypogonadism and to check whether certain questionnaires can be a simple screening tool for hypogonadism in this setting.

## Materials and methods

### Patient population

In this monocentric study we identified 402 patients who received a systematic therapy for GCT at the Department the West German Tumor Center (WTC) of the University Hospital Essen, Germany from 2001 until 2019. The study was approved by the local ethics committee (21-9860-BO) and conducted in accordance with the ethical standards of the Declaration of Helsinki. All prospective patients gave written informed consent prior to study inclusion.

All patients were offered a post therapeutic-hormone status check by blood sampling as well as answering two standardized questionnaires (International Index of Erectile Function (IIEF-5) and The aging males’ symptoms rating scale (AMS)) to record symptoms of testosterone deficiency. If no response was received, a second invitation was sent. A vital status search was initiated via the WTC for all identified patients.

### Germ cell tumor chemotherapy

We evaluated the GCT-associated systemic therapies performed in all patients. We collected data of pre-systemic local therapy (orchiectomy unilaterally or bilaterally), adjuvant therapy, first-line therapy or advanced systemic therapy lines and also retroperitoneal lymph node dissection (RPLND). Analyses sets were grouped according to their exposure to chemotherapy into 3 groups: Patients who received a maximum of 2 cycles of chemotherapy, patients who completed 3 to 4 cycles and third, additional lines of therapy, e.g. at least 5 cycles.

### Patient reported outcomes

The IIEF-5 is a validated tool to detect erectile dysfunction, which is among others a functional consequence of testosterone deficiency but may also detect other causes^13–15^. The questions are graded on a 5-point scale. The maximum potential score was 25, with a higher score indicating better sexual function. Patients with an IIEF-5 score ≥ 22 were deemed fully functional, patients ≤ 21 were labelled as impaired with possible symptoms of hypogonadism.

The second questionnaire implemented in our study was the AMS^16^. This questionnaire is an established tool for the assessment of symptoms of low testosterone levels and detection of late-onset hypogonadism. Although developed and validated in an elderly patient population, the questions relate to generic signs and symptoms of hypogonadism and therefore was considered appropriate in our younger patient population, as it has already been performed under other research topics as well^17–20^. The AMS questions are graded on a 5-point scale. Patients were classified as per their psychological, somatic and sexual function. The maximum score was 85, with a higher score indicating worse function. Patients with an AMS score ≤ 26 were labelled as having no signs of symptomatic hypogonadism, patients with a score at least 27 were labelled as having symptoms of hypogonadism.

### Evaluation of the hormonal status

We determined luteinizing hormone (LH), follicle-stimulating hormone (FSH) and testosterone in the blood^6^. Blood sample was taken in the morning between nine and eleven o’clock considering circadian rhythm. Latent hypogonadism was defined as serum testosterone levels of 8–12 nmol/l, whereas a manifest hypogonadism was defined as serum testosterone levels < 8 nmol/l.

In case of an isolated LH elevation (normal value 1–8 IU/L) with normal T-levels, we interpreted this as an indication of Leydig cell damage. In case of an isolated FSH elevation (normal value 1–10 IU/L), this was interpreted as an indication of Sertoli cell damage. In these cases, the questionnaires were evaluated with regard to symptomatology and the patients were informed about this.

### Statistical analysis

Continuous variables and scores of the questionnaires are expressed as median and interquartile range. Statistical analyses were performed using R version 4.2.1 (R Foundation for Statistical Computing, Vienna, Austria).

## Results

### Patient characteristics

Initially, 138 invitations were not deliverable. After vital status research, in total 198 patients (49%) did not respond to our invitation at all, only 1% explicitly declined to participate. The participation rate was 8.1% for up to 2 chemotherapy (CTx) cycles, 11% for 3–4 cycles and 18% for 5 cycles, indicating a rising willingness to participate with more cycles (Table 1).

In total, 44 patients (11%) participated in the study and were therefore included in the final analysis. From these, 32 (73%) also appeared for blood sampling. 32% (*n* = 14) received up to 2 courses as adjuvant therapy, 17 patients (39%) 3–4 courses as completed first line therapy, 13 patients (29%) a salvage therapy (shown in Table 2). The majority of patients (*n* = 41, 93%) had also received prior unilateral orchiectomy. 2 patients (5%) received bilateral orchiectomy (due to bilateral tumor or atrophy, one each). Three patients (7%) received primary extragonadal resection. Furthermore, 11 patients (25%) underwent RPLND (Table 2). The median duration of the last chemotherapy cycle until follow up was 159 months (Table 2).

**Table 1:** Response rate.

	Total cohort (*n* = 402)	≤ 2 courses CTx (*n* = 172)	3–4 courses CTx (*n* = 151)	≥ 4 courses CTx (*n* = 72)	Not applicable (*n* = 7)
Initial interest in study participation	79 (20%)	30 (17%)	32 (21%)	16 (22%)	1 (14%)
Undeliverable	138 (34%)	71 (41%)	47 (31%)	20 (28%)	3 (43%)
No response at all	198 (49%)	77 (45%)	79 (52%)	38 (53%)	4 (57%)
Rejection	4 (1%)	3 (1,7%)	0	1 (1%)	0
Actual participation	44 (11%)	14 (8.1%)	17 (11%)	13 (18%)	0

Percentage is given in parenthesis. Patients can be in several groups, so that the sum per column does not always add up to 1 or 100%.

**Table 2:** Patient baseline characteristics.

Patient characteristics	Total cohort (*n* = 44)
Age at initial diagnose, median [years]	35 [29–41]
Follow up after last cycle of CTx [months]	159 [94–191]
Previous therapies	
Orchiectomy unilateral, n	39 (89%)
Orchiectomy bilateral, n	2 (4.5%)
Extragonadal resection, n	3 (6.8%)
Platinum-therapy	
≤ 2 courses, n	14 (32%)
3–4 courses, n	17 (39%)
Salvage CTx, n	13 (29%)
RPLND	11 (25%)

Percentage is given in parenthesis, the interquartile range [Q1-Q3] in brackets.

### Patient reported outcomes

The IIEF-5 was completed by 42 patients. In our study, half of the patients (50%) had abnormal values. The proportion between the three groups (chemotherapy ≤ 2 courses, 3–4 courses within first line therapy and salvage-chemotherapy) was nearly balanced: 43% of the first group showed abnormal values, while it was 44% in the group with first line treatment and 67% with at least 5 courses (Table 3).

43 patients answered the AMS. From these, more than half (63%) showed suspicious values defined as ≥ 27 pts.). In this questionnaire, the proportion of affected patients was comparatively larger both between the individual groups and compared to the relations regarding IIEF-questionnaire (CTx ≤ 2 courses: 50% vs. 3–4 courses 63% vs. salvage CTx 77%) (*p* = 0.387) (Table 3).

**Table 3:** Questionnaires.

Patient characteristics	Total cohort (*n* = 43)	≤ 2 courses CTx (*n* = 14)	3–4 courses CTx (*n* = 16)	Salvage CTx (*n* = 13, resp. 12)
AMS (*n* = 43)				
Suspicious value	27 (63%)	7 (50%)	10 (63%)	10 (77%)
Normal value	16 (37%)	7 (50%)	6 (37%)	3 (23%)
IIEF-5 (*n* = 42)				
Suspicious value	21 (50%)	6 (43%)	7 (44%)	8 (67%)
Normal value	21 (50%)	8 (57%)	9 (56%)	4 (33%)

Percentage is given in parenthesis. One missing dataset of questionnaires in the group with 3–4 courses, therefore only 43 instead of 44 patients. In the salvage group one participant did not fill in the IIEF questionnaire either.

### Hypogonadism

In total, from 44 patients 32 (72.7%) patients participated in the blood draw, values were available for *n* = 30 (LH), *n* = 29 (FSH) and *n* = 31 (testosterone) patients. The reference values and the distribution of the abnormal values are shown in tabular form (shown in Table 4). Latent hypogonadism (serum testosterone 8–12 nmol/L) was found in *n* = 9 (29.0%) and manifest hypogonadism (testosterone < 8 nmol/L) in *n* = 8 (25.8%) patients. Proportionally, manifest hypogonadism was diagnosed more frequently in most subgroups (*p* = 0.17) (Table 4).

**Table 4:** Blood parameters.

Blood parameters	Total cohort (*n* = 44, respectively 32)	≤ 2 courses CTx (*n* = 14, resp. 7)	3–4 courses CTx (*n* = 17, resp. 14)	Salvage CTx (*n* = 13, resp. 11)
Blood parameters				
FSH [U/l], *n* = 29	16.2 [12.3–21.7]	12.7 [7.9–23.8]	15.2 [13.1–18.8]	15.4 [11.8–22.2]
LH [U/l], *n* = 30	6.4 [4.8-8.0]	6.1 [5.1–7.8]	5.2 [4.3–6.5]	7.2 [6.4–7.9]
Testosterone[nmol/l], *n* = 31	11.3 [8.3–17.3]	8.5 [7.2–12.1]	13.6 [10.8–19]	7.9 [2.0–15.0]
Detected hypogonadism, *n* = 31				
Latent hypogonadism	9 (28%)	2 (29%)	5 (36%)	2 (18%)
Manifest hypogonadism	8 (25%)	3 (43%)	1 (7%)	4 (36%)

95% confidence interval is given in parenthesis, the interquartile range [Q1-Q3] in brackets. From the total cohort, complete blood values were only available for 29 participants. In addition, only the FSH value was available from one additional participant, only the testosterone value from 2 further additional patients. Latent hypogonadism was defined as serum-testosterone 8–12 nmol/l, manifest hypogonadism as serum-testosterone < 8 nmol/l. In the adjuvant group, values of testosterone were available for 7 patients, of FSH and LH for 6 patients. In the group of primary therapy, values of testosterone and FSH were available from 13 patients, of LH from 14 patients. In the salvage group values of testosterone were available for 11 patients, for FSH and LH from 10 patients.

### Association of questionnaires and blood samples

A further analysis with a sub cohort (*n* = 30), of which both questionnaire and blood values were available (AMS *n* = 30 IIEF-5 *n* = 29), revealed the percentage of patients with suspicious serum testosterone levels and symptomatic burden: In the AMS group, 12 patients (63%) with suspicious scores also reported symptoms, which was a bit higher than in the IIEF-5 group. Here, from 15 symptomatically affected patients 9 (60%) had low serum testosterone levels. In this context, 4 patients (36%) in the AMS and 6 (43%) in the IIEF-5 group had normal values in the questionnaires but low serum testosterone levels. For detailed information regarding the sub-cohorts see Table 5.

We further evaluated which patients of the sub cohort, of which a complete data set was available, were already under hormone substitution with testosterone: 3 patients with pre-existing substitution had manifest hypogonadism as well as abnormal values of both AMS and IIEF-5. Another patient was serologically in the range of latent hypogonadism and also reported symptom burden in both questionnaires.

**Table 5:** Questionnaires of the cohort with available blood parameters.

Patient characteristics	Total cohort (*n* = 30 btw. 29)	≤ 2 courses CTx (*n* = 7)	3–4 courses CTx (*n* = 12)	Salvage CTx (*n* = 11, resp. 10)
AMS (*n* = 30)				
Suspicious value	19 (63%)	3 (43%)	9 (75%)	7 (64%)
Hereof low serum testosterone	12 (63%)	3 (100%)	5 (56%)	4 (57%)
Normal value	11 (37%)	4 (57%)	3 (25%)	4 (36%)
Hereof low serum testosterone	4 (36%)	2 (50%)	0	2 (50%)
IIEF-25 (*n* = 29)				
Suspicious value	15 (52%)	3 (43%)	7 (58%)	5 (50%)
Hereof low serum testosterone	9 (60%)	3 (100%)	4 (57%)	2 (40%)
Normal value	14 (48%)	4 (57%)	5 (42%)	5 (50%)
Hereof low serum testosterone	6 (43%)	2 (50%)	1 (20%)	3 (60%)

Percentage is given in parenthesis. Low serum testosterone was defined as < 12 nmol/l.

30 patients were with available data for AMS and testosterone, 29 patients with available data for IIEF and testosterone (in the group of salvage therapy one patient did not answer the IIEF-5).

## Discussion

The impact of chemotherapy in GCT therapy is known to attenuate long-term consequences like hypogonadism, which has a high impact on the quality of life of the patients. Guidelines recommend assessing symptoms and determining testosterone and LH levels as part of the follow-up^6,21^.

The rate of hypogonadism in our patients is consistent with data from the literature, which indicated that patients who underwent surgery and chemotherapy had a higher incidence of lower serum testosterone than patients with surgery alone^12,22^. In our study, 17 patients (53.1%) hadlow serum testosterone levels, from these 8 (25.0%) had a manifest hypogonadism. Previously reported retrospective studies have shown that 11–16% of GCT survivors have subnormal serum testosterone levels on long-term follow-up (> 5 years after treatment).

Up to 23% patients with low testosterone levels reported a decreased quality of life^5^. In our study, a high percentage of patients reported symptom affection in the questionnaires: 63% in the AMS and 50% in IIEF-5 questionnaire. Importantly, we further recognized a high rate of patients with abnormal values in the questionnaires demonstrating impaired quality of life and hypogonadism in serum blood samples. By the use of the AMS, 12 out of 19 (63%) patients with suspicious values in the questionnaire were identified having hypogonadism in serum blood sample. The detection rate of the IIEF-5 was comparable high (9 from 15 patients, 60%). Most of these patients were undiagnosed with hypogonadism and were therefore not under therapy in the moment of study participation. Nevertheless, patients identified in our study were predominant symptomatic and medical treatment was indicated. Important to mention in this context are also the patients with pre-existing substitution but still manifest (3 patients) or latent (1 patient) hypogonadism as well as abnormal values of both AMS and IIEF-5. Here, an insufficient substitution can be assumed and therapy optimization was offered.

Although these results indicate the principal utility of IIEF-5 and AMS to detect endocrine malfunction during follow-up in younger male patients, it may also indicate that our study enriched for symptomatic patients, because such patients were more likely to respond to our study invitation. Guidelines suggest to treat patients with hypogonadism only if they are symptomatic^6,21^. Our data indicate that routine assessment of hormone replacement therapy is mandatory for identification and successful treatment. While adherence to guidelines is difficult to assess in our study, the medical need in this young patient population is evident and consequent and increased screening for symptomatic gonadal dysfunction should be incorporated in routine care of cancer survivors of GCT. We assume that the high rate of conspicuous patients could be due to the long follow-up intervals and patients avoiding follow-up care itself. Therefore, putative measures to increase awareness are information about signs and symptoms and possible treatments of male endocrine dysfunction should be provided.

A high proportion of patients showed abnormalities in the questionnaires, but only 17 had a laboratory hypogonadism detected. Therefore, the question arises how exactly the detection should be implemented with the aim of avoiding over- and underdiagnosis. The strength of the questionnaires is its functional assessment that includes also other causes than hypogonadism and therefore should trigger further investigation if hormone levels remain within normal limits. This could lead to detection of additional chemotherapy associated adverse events, such as cardiovascular diseases. Early detection and intervention could have positive effects on the individual health status and therefore lead to additional benefits beyond hormone replacement in patients with chemotherapy exposure. In addition, these questionnaires offer objectifiable, standardized question complexes that can be easily integrated into everyday practice.

Unfortunately, due to the low response rate of 7–10% it was not possible to generate a valid statement about the effect on hormone status depending on the respective chemotherapy regimen, despite the low-threshold offer of hormone status testing. Assuming that a similar proportion of patients evade long-term follow-up for long-term toxicities, the rate of undiagnosed hypogonadism could be very high. Therefore, it is important to create awareness among and identify hurdles in the care of these patients as well as to develop easy detection methods that can be implemented in guidelines. The reasons for the loss of patients after initial therapy are complex and confounding factors should be identified to improve implementation of guideline-based diagnosis and therapy.

The point of the extremely poor response and participation rate deserves special consideration in the context of this study. The reasons are complex and remain speculative in our study. On the one hand, patients with testicular tumors are often affected at a very young age. In the course of their life a higher mobility for study or a job must be assumed than in adult patients. For this reason, we were unable to obtain any contact or address data at this point in 138 patients. Another important point is the right to forget. Young patients, if they are affected by a disease, are often more psychologically burdened than older patients^23^. The young patients in this group may want to come to terms with the disease or have come to terms with it and do not want to be confronted with it again in order to avoid bringing up the psychological stress of the past^24^. Nevertheless, studies postulated that survivors of germ cell cancer experience a similar quality of life compared to other men the same age^23,25,26^. Another reason for the lack of response could be that most patients feel well cared for, well followed up and also connected by the urologists in their own practice, so that there is no desire for further checks. Clearly, more research is required to understand the burden of care and possible barriers in order to improve outcomes.

In this context, Cancer Survivorship Programs should be mentioned, which are intended to help those affected to counteract long-term physical toxicities, among other things, but also to deal with the psychosocial problems and specific support needs after the testicular tumor disease adapted to the individual life situation. In addition to multidisciplinary medical services, survivorship programs include psycho-oncological care, socio-legal counselling, music or art therapy or yoga, adapted sports and exercise programs, nutrition and life-style^27^. This assumption might be confirmed by the fact that in a Danish study with a different organisation of health care system the participation rate was 80%. The patients here expressed a high level of satisfaction in general regarding the follow-up. Another point that should be mentioned is that the study was conducted during the Corona pandemic and therefore willingness to interact with others was limited due to e.g. home office. Studies found, that factors like own safety, inconvenience, accessibility and the lack of benefit limited participation during COVID-19 pandemic, with an increased focus on the environment^28^. As our study visits took place in the outpatient department in our hospital, this might have impaired the rate of participation, too.

Our study has limitations. The major limitation was the low participation and the possible selection for symptomatic patients. As described in detail above, this limited the interpretation and analysis of our data. However, screening for gonadal dysfunction by questionnaires was feasible and supports further research for GCT cancer survivors. Due to size and heterogeneity of our data, the use of p-values has limitations and does not reach no significance.

## Conclusion

Hypogonadism is an important and common issue among testicular tumor patients, which requires detection and adequate therapy. Our study indicated the difficulties of today’s research in a young patient population. Further studies should assess barriers for study participations to identify patient’s needs to customize patient’s care.

## Data Availability

The data that support the findings of this study are available from the corresponding author, AH, upon reasonable request.
